# Collective cell migration: Implications for wound healing and cancer invasion

**DOI:** 10.4103/2321-3868.113331

**Published:** 2015-06-26

**Authors:** Li Li, Yong He, Min Zhao, Jianxin Jiang

**Affiliations:** 1 Department of Respiratory Diseases, Daping Hospital, Third Military Medical University, Chongqing, 400042; 2 State Key Laboratory of Trauma, Burns and Combined Injury, Institute of Surgery Research, Daping Hospital, Third Military Medical University, No.10 Changjiang Branch Road, Daping Main Street, Yuzhong District, Chongqing, 400042 P. R. China; 3 Department of Dermatology, Institute for Regenerative Cures, University of California, Davis, CA 95817 USA

**Keywords:** Wound healing, cancer invasion, collective migration, E-cadherin, mechanical force Introduction

## Abstract

During embryonic morphogenesis, wound repair and cancer invasion, cells often migrate collectively via tight cell-cell junctions, a process named collective migration. During such migration, cells move as coherent groups, large cell sheets, strands or tubes rather than individually. One unexpected finding regarding collective cell migration is that being a “multicellular structure” enables cells to better respond to chemical and physical cues, when compared with isolated cells. This is important because epithelial cells heal wounds via the migration of large sheets of cells with tight intercellular connections. Recent studies have gained some mechanistic insights that will benefit the clinical understanding of wound healing in general. In this review, we will briefly introduce the role of collective cell migration in wound healing, regeneration and cancer invasion and discuss its underlying mechanisms as well as implications for wound healing.

## Introduction

In essential physiological processes including morphogenesis, wound healing and tissue regeneration, cells often move as a tightly or loosely associated cohesive group. This type of migration, during which cells are influenced by the interaction with their neighbors, is known as ‘collective cell migration’. Collective cell migration is now recognized as a hallmark of tissue-remodeling events. Indeed, virtually all living tissue is constructed by collective cell migration, which plays an important role in the initial symmetry-breaking and leader-follower organization of cell groups during embryonic development.[1] In the zebrafish lateral line primordium, a migrating cluster of approximately two hundred cells migrate, grow, divide and differentiate simultaneously and assemble into a series of connected epithelial rosette-like mechanosensory organs.[2] During oogenesis in *D. melanogaster*, a tightly packed cluster of border cells migrates together and presents a distinct collective mechanism of polarity and guidance.[3] Lumenal epithelial cells elongate collectively within elongating ducts during branching morphogenesis in the mammary gland.[4] Collective migration has also been recognized as an important mechanism for wound healing and cancer invasion (see details below).

**Table 1:** 

Access this article online	
**Quick Response Code**:	**Website**: www.burnstrauma.com
	**DOI**: 10.4103/2321-3868.113331

When cells in a group interact with the same chemotactic and mechanical signals as those of isolated cells, they may respond differently in a collective fashion. The junctions between cells maintain “supracellular” structures and result in collective polarization, intercellular force generation, decision-making and eventually, tissue organization. Recent studies discovered that cells of the same type respond differently to the same directional cues when isolated versus when acting as a cohesive group.

## Cells acting collectively respond differently from cells in isolation to environmental signals

**Figure 1:**
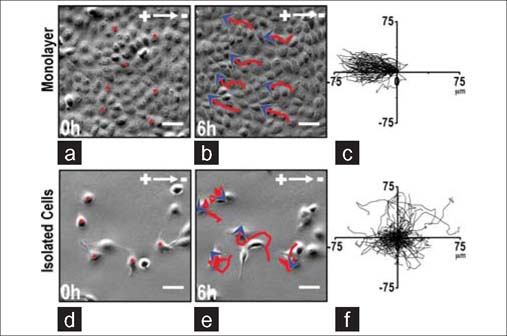
Robust electrotaxis in monolayer, not in isolation. (a-c) MDCK II cells in a sheet showed robust collective electrotaxis in an EF of 200 mV/mm for 6 hours. Red lines with blue arrowheads represent migration paths and direction. (c) Cell migration trajectories with starting positions placed at the origin. (d–f) In a field of the same strength, isolated MDCK II cells did not show electrotaxis. Scale bars, 50 μm. Figures obtained from published paper by the author: *CMLS 2012, 69(16):2779–2789*.

**Figure 2:**
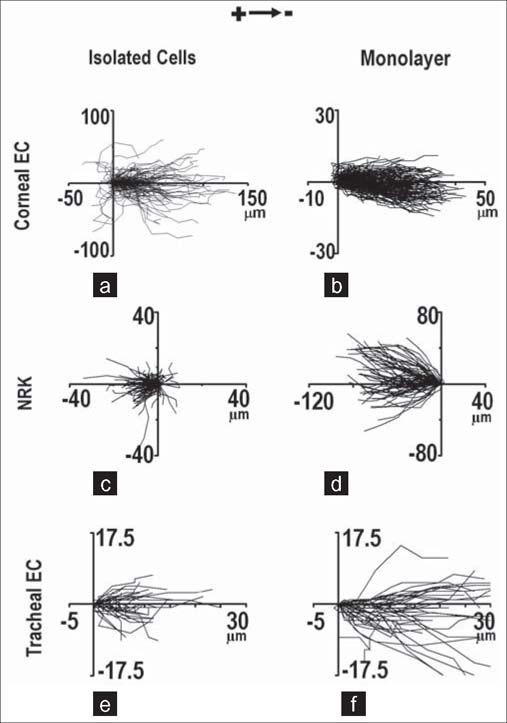
Enhanced collective electrotaxis than in isolation of various cell types. (a, b) Migration tracks Corneal epithelial cells in isolation and in monolayer in an EF of 200 mV/mm for 6 hours. (c, d) Migration tracks of NRK cells in isolation and in monolayer in an EF of 200 mV/mm for 2 hours. (e, f) Migration tracks of tracheal epithelial cells in isolation and in monolayer in an EF of 200 mV/mm for 30 minutes. Figures obtained from published paper by the author: *CMLS 2012, 69(16):2779–2789*.

It is important to understand whether cells acting collectively behave as a simple collection of single cells or have additional properties in terms of movement, guidance, force generation or signal transduction. Cells acting collectively may respond to the same signal differently compared to isolated cells. Several studies have introduced this striking difference. A subset of *Xenopus* neural crest (NC) cells respond efficiently to the chemoattractant SDF1, whereas isolated cells barely respond.[5] In isolated cells that do not interact with other cells and have unstable cell polarity, the chemoattractant SDF1 is unable to induce a distinct anterior-posterior polarity, and the cells chemotax poorly. With an increase in cell density, the cells are able to interact with each other, and anterior-posterior polarity is established due to contact inhibition of locomotion at sites of interaction between cells. Under this condition, these well-polarized cells respond much better to the same chemoattractant with more stabilized protrusions.[5] Similar to the improved collective response in chemotaxis, our group has reported a group of size-dependent directional migratory response of cells undergoing electrotaxis.[6] In the commonly used epithelial cell line MDCK, cells in a monolayer migrated directionally to the anode in an electric field (EF), whereas isolated cells displayed random migration in an EF of the same strength [Figure 1]. In that study, we also confirmed the better collective electrotaxis response in several other types of epithelial cells, including normal rat kidney cells, bovine corneal epithelial cells and monkey tracheal epithelial cells [Figure 2]. Traction forces, which play an important role in cell migration, also occurred in different patterns in single cells compared with the cell monolayer. For a single cell, traction forces were generated by the attachment of the actin network to the substrate at the leading edge of the cell, whereas at the trailing edge, the forces were a result of the actin network slipping over the substrate.[7,8] For epithelial sheets, the traction forces are mainly generated at the edges and cell-cell junctions.[9] This different pattern of traction force distribution may be a result of the mechanical communication between cells and may play a role in the collective cell migration of epithelial sheets.[10]

## Collective cell migration in wound healing and regeneration

**Figure 3:**
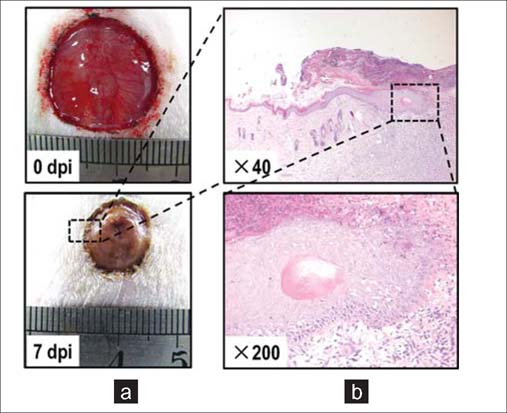
Collective migration of epithelial sheets in skin wound healing. (a) Healing process of a skin wound with diameter of 2cm on the rat back. Dpi,days post injury. (b) Histopathology of skin wound on 7dpi. Epithelial sheets proliferate and migrate collectively and directinally into the wound center.

Collective migration is one of the hallmarks of wound healing. During wound healing, epithelial cells migrate collectively as a coherent sheet to heal wounds. Wounding an epithelial monolayer induces directional migration of a cell sheet, which maintains tight intercellular adhesion.[11,13] In multilayered corneal epithelium and rat skin, cells in the stratified epithelium also migrate en masse following injury[14,16] As shown in Figure 3, during skin wound healing, epithelial cells proliferate and migrate collectively into the center of the wound. In corneal wounds, cell-cell junction molecules, such as the tight junction-specific protein occludin, are present beginning one cell layer from the leading edge through to the posterior cells.[17] Using 3D time-lapse analysis, we were able to track the movement of individual cells in the multi-layered epithelium in corneal wounds. Quantitative analysis demonstrated that over 95% of the cells moved at similar migration speeds and trajectories with very little change in their relative position.[18] The collective migration, maintaining intercellular connection and relative positions are conserved in the wound healing of many types of epithelia, such as cornea, skin, respiratory and digestive epithelia, and endothelium.[12,13,19]

Vessel sprouting, which occurs during restoration of functional multicellular organs during tissue repair, requires the regenerative collective migration of endothelial cells. During regeneration, a network of new vessels is formed by collective strands of endothelial cells penetrating a provisional fibronectin-rich wound matrix.[20,21] These endothelial sprouts are guided by highly polarized tip cells that protrude long actin-rich filopodia and sense the local high concentration of vascular endothelial growth factor-A (VEGF-A) secreted by astrocytes. The tip cell is followed by a multicellular stalk of endothelial cells, which are connected by VE-cadherin and form an inner lumen.[22,23] By these means, extracellular VEGF gradients control the directional collective migration of endothelial cells in angiogenesis.

## Collective cell migration in tumor metastasis

Cancer metastasis, the spread of cancer cells to distant organs, accounts for many cancer-related deaths. Migration is one of many factors implicated in this phenomenon. Collective cell migration plays a role not only in development, wound healing and regeneration but also in cancer metastasis. Increasing evidence indicates that clusters of metastatic cells invade collectively in the vasculature and lymphatics of cancer patients.[24,25] Many types of epithelial cancers, including oral squamous cell carcinoma, breast cancer and colorectal carcinoma, predominantly exhibit collective invasion in cultures.[26,27] Several types of epithelial cancers display features of collective invasion in histopathological sections, including intact cell-cell junctions as well as the expression of E-cadherins and other adhesion molecules in tumor regions deep inside the normal stroma.[1,28,29] In histopathological sections of several types of epithelial cancers, the primary tumor is surrounded by secondary cancer cells in the form of clusters, chains or sheets.[27]

## Control of collective cell migration

Understanding the foundation of collective migration, in contrast to that of single cell movement, will facilitate the development of strategies that may either suppress or enhance collective movement in a defined manner. How cells cooperate and migrate collectively as a whole is not fully understood. The currently known underlying cellular and molecular mechanisms of collective migration include cell-cell adhesion, force generation and orientation, collective cell polarization and collective guidance by chemical and mechanical signals, which are discussed below.

### Cadherin-based cell-cell adhesion

The maintenance of stable physical links between cells plays an essential role in collective migration. Cells in epithelial sheets are connected together by cell-cell adhesion molecules, including members of the cadherin family and other transmembrane proteins of the immunoglobulin superfamily. E-cadherin forms calcium-dependent homophilic intercellular adhesions between epithelial cells. Lumenal epithelial cells retain E-cadherin along cell-cell interfaces while elongating collectively within elongating ducts during branching morphogenesis in the mammary gland.[4] Blocking E-cadherin with specific antibodies causes disruption of coordinated cell movement in epithelial wounds, which results in a ragged, uneven epithelial wound margin.[17] In addition, we have reported that E-cadherin plays an essential role in the directional migration of large epithelial sheets, in which E-cadherin mechanically coordinates individual cells in cell sheets and thus promotes collective electrotaxis.[6] Other cadherins, including neural cadherin (N-cadherin) and vascular endothelial cadherin (VE-cadherin), are also implicated in collective migration. For example, the chemotaxis of neural crest cells was recently demonstrated to require N-cadherin.[5]

### Generation and orientation of mechanical stresses in collective cell migration

Mechanical stress exerted at cell-cell and cell-substrate interfacial boundaries is involved in a variety of physiological processes, including cell migration. During collective cell migration, each individual cell is physically constrained by its neighbors and thus experiences multiple stresses, including normal stress and shear stress. Normal stress is oriented perpendicularly and shear stress is oriented parallel to the cell-cell contact surface.[30] Through mapping of normal stresses and shear stresses cells exerted on a gel substrate, and correlating them with cellular migratory directions, Dhananjay *et al*, reported a novel mechanism that controls collective migration. Neighboring epithelial cells join forces to transmit appreciable normal stress across the cell-cell junction but migrate along orientations of minimal intercellular shear stress.[31] We also reported that during directional collective migration of large epithelial sheets, the traction forces that leading edge cells exert on the substrate displayed a significant orientation, indicating that the orientation of traction forces plays an important role in collective guidance.

### Collective polarization: “leading cells” in collective cell migration

During collective migration of an epithelial monolayer to a model wound, active “leader cells” manifest with a non-epithelial fibroblast-like appearance and protrude filopodia, thus leading the migration of the cell monolayer.[11] The “fingers” of the border contain a pluricellular subcortical actin belt, indicating strong mechanical tensions and signaling between the leader cells and its followers.[11] Moreover, the successful formation of leader cells is dependent on the spatial and temporal coordination of RhoA activity across the epithelial edge. Constitutive activation of RhoA suppresses the formation of leader cells, whereas RhoA inhibition results in most edge cells transforming into leader-like cells.[2] As discussed above, during angiogenic sprouting, which is a collective migration process, specialized vascular endothelial tip cells sense attractive and repulsive cues at vascular fronts and then determine the behaviors of neovascular sprouting. Several key molecules have been reported to control endothelial tip cell functioning. VEGF induces tip cell filopodia growth, thus guiding the direction of vascular sprouting.[32] SDF1/CXCR4 activates tip cells and then promotes angiogenesis in retina.[33] Additionally, delta-like 4 (Dll4)-notch signaling determines the fate of both endothelial tip cells and their followers, which are called stalk cells.[23]

## Perspective and clinical relevance: Why collective migration is important in wound healing

A better understanding of collective cell migration is of clinical relevance. In wound healing, the primary goal for epithelial cells is to restore the epithelial barrier. It is therefore important that while the epithelial cells migrate over the wound bed, proper cell-cell adhesion is maintained so that the epithelial barrier is not further compromised. The application of most growth factors or other migration-stimulating chemicals usually has the potential detrimental effect of “scattering” cells in epithelial sheets, thus compromising epithelial barrier function. Epidermal growth factors (EGFs), for example, induce barrier function decrease and the dispersal of cells.[34] Growth factors that stimulate cell migration break down cell-cell junctions, thereby dispersing epithelial sheets and are therefore named “scatter factors”.[35] Therefore, in wound healing management, therapeutic approaches based on the application of factors should pay attention to the possible dispersal effects on the collective migration of epithelial sheets. On the other hand, epithelial sheets with strong cell-cell adhesion respond significantly better to physical stimulation, such as electrical cues. These directional cues may be further developed with powerful tools to promote wound healing. For example, drugs that can enhance wound-induced endogenous EFs may be incorporated into a bandage that can be applied onto skin wounds. Those drugs can then constantly exert effects on epithelial cells of local skin wound edges and may promote the collective migration of epithelial sheets to heal wounds. Indeed, our initial experiments on rat skin wound healing produced promising results, showing that the enhancement of skin wound EFs by applying prostaglantin E2 (PGE2) promotes skin wound healing.[36]

A variety of fundamental processes in development, health and disease depend on collective cellular migration. Despite some molecular mechanistic insights so far, our understanding of collective migration lacks predictive power and remains largely descriptive. Some of the unsolved questions are listed here. First, it remains unclear how various chemical, mechanical and, potentially, electrical signals from surrounding tissue and neighboring cells become integrated to allow coordinated multicellular movement. Second, it remains unclear whether “leader cells” exist to lead the cell sheets in directional collective migration. As discussed above, several studies emphasized the leading cell mechanism in collective migration. However, the finding that traction forces that drive collective migration manifest predominately many cell rows posterior to the leading front edge and extend across enormous distances indicates that “leader cells” may not truly be “leaders” but are pushed forward by the cells behind them.[37] Third, it remains unclear why cells that act collectively respond better to the same signal than cells that act individually. One possibility is that the weak- and better-responding cells are maintained together by stable cell-cell junctions, thus averaging differences among cells. More detailed mathematical analysis and modeling are required to clarify the pattern of collective cell migration.

In summary, collective cell migration is increasingly recognized as a common theme that links morphogenesis, regeneration and cancer biology. Understanding the common rules of collective cell migration will lead to the development of strategies that either enhance or suppress collective movement in a defined manner.
